# Steady-State Pharmacokinetics of Intravenous Hydromorphone in Japanese Patients With Renal Impairment and Cancer Pain

**DOI:** 10.1089/jpm.2022.0289

**Published:** 2023-06-02

**Authors:** Toshihiko Nakatani, Kazuhito Shiosakai, Tatsuya Hashimoto, Masao Shionoya, Takaaki Akasaka, Kaoru Toyama, Hitoshi Ishizuka, Yoji Saito

**Affiliations:** ^1^ Department of Palliative Care, Shimane University Faculty of Medicine, Izumo, Japan.; ^2^ Data Intelligence Department, Daiichi Sankyo Co., Ltd., Tokyo, Japan.; ^3^ Department of Anesthesiology, Palliative Care Center, Shimane University Hospital, Izumo, Japan.; ^4^ Statistical Analysis Department, Mebix, Inc., Tokyo, Japan.; ^5^ Primary Medical Science Department, and Daiichi Sankyo Co., Ltd., Tokyo, Japan.; ^6^ Quantitative Clinical Pharmacology Department, Daiichi Sankyo Co., Ltd., Tokyo, Japan.; ^7^ Department of Anesthesiology, Shimane University Faculty of Medicine, Izumo, Japan.

**Keywords:** cancer, hydromorphone, pain, pharmacokinetics, renal impairment

## Abstract

**Background:**

The opioid analgesic hydromorphone has a low renal excretion ratio; however, exposure after oral administration is several times higher in those with moderate or severe renal impairment.

**Objectives:**

We evaluated the impact of renal impairment on the steady-state pharmacokinetics of intravenously administered hydromorphone in patients with cancer being treated for pain.

**Design:**

This was an open-label, prospective, parallel-comparison, interventional clinical pharmacology study.

**Setting/Subjects:**

This study was conducted at one hospital in Japan. Using creatinine clearance (CLcr) values, patients were grouped according to kidney function: CLcr ≥90 mL/min (normal), 60–<90 mL/min (mild impairment), 30–<60 mL/min (moderate impairment), or <30 mL/min (severe impairment).

**Measurements:**

Hydromorphone was administered by constant infusion to patients at the same constant dose rate as at the time of enrollment. Hydromorphone and its glucuronide metabolite concentrations in plasma and urine were measured by liquid chromatography-mass spectrometry. Pharmacokinetic parameters at steady state were assessed using noncompartmental analysis.

**Results:**

Thirty-two patients were enrolled (normal, *n* = 3; mild, *n* = 10; moderate, *n* = 15; and severe, *n* = 4). Adjusted geometric mean ratios for hydromorphone steady-state clearance (CLss) for patients with impaired versus normal renal function were 0.69 (90% confidence interval [CI], 0.41–1.14), 0.52 (90% CI, 0.31–0.84), and 0.55 (90% CI, 0.30–1.02) for mild, moderate, or severe impairment, respectively. Exposures to the metabolite hydromorphone-3-glucuronide generally increased with renal impairment. No adverse event was reported.

**Conclusion:**

Hydromorphone CLss in patients with impaired renal function (moderate and severe) was decreased ∼50% of that of normal renal function.

## Introduction

Around one-third of cancer patients experience cancer pain, which severely impacts quality of life.^1^ In patients with advanced cancer, the percentage is higher (66%–71%).^2,3^ Guidance from the World Health Organization regarding analgesics for cancer pain management emphasizes the importance of selecting an appropriate dose for each individual patient.^4^ Careful consideration and a good understanding of analgesics are critical when making such decisions.

Hydromorphone hydrochloride (hydromorphone) has a higher affinity for opioid receptors and a fivefold to eightfold stronger analgesic effect than morphine.^5–7^ Hydromorphone is recommended as an alternative to morphine in guidelines^8–11^ and is widely used in cancer pain management.^12^

Morphine clearance is similar in patients with normal and impaired renal function.^13^ However, morphine glucuronide metabolites are excreted renally and accumulate in patients with renal dysfunction. Accumulation of the metabolite morphine-6-gluconuride can result in central nervous system effects. Thus, morphine use is not recommended for patients with severely reduced renal function.^13,14^

In the case of hydromorphone, only a small amount of the hydromorphone dose is excreted unchanged in the urine.^15,16^ The major metabolite of hydromorphone is hydromorphone-3-glucuronide (H3G), formed by UDP-glucuronosyltransferase-2B7 (UGT2B7).^13,17^ H3G has minimal pharmacological activity. A clinical pharmacology study of oral hydromorphone in volunteers has shown that the area under the curve (AUC) is twofold to fourfold greater in patients with moderate (defined as creatinine clearance [CLcr] 40–60 mL/min) or severe (defined as CLcr <30 mL/min) renal impairment, compared with subjects with normal renal function (defined as CLcr >80 mL/min).^18^ Prescribing information state that patients with moderate renal impairment should be started on a lower dose, with even lower starting doses for patients with severe renal impairment.^19–21^

However, this information is based on data derived mainly from oral doses of hydromorphone and, to date, there has been no detailed investigation of the steady-state pharmacokinetics of hydromorphone after constant intravenous infusion in cancer patients with renal impairment. Therefore, our study aimed to understand whether and how renal function influence the pharmacokinetics of hydromorphone and its major metabolite, H3G, after constant intravenous infusion in patients with cancer, who had normal renal function or different degrees of renal impairment. In addition, we investigated the influence of uremic toxins on the pharmacokinetics of hydromorphone as an exploratory endpoint.

## Materials and Methods

### Patients

Inpatients with cancer, ≥20 years of age, who had been under pain control and were receiving a continuous infusion of hydromorphone (fixed dose) for ≥72 consecutive hours were eligible for inclusion in this study. Rescue dosing of hydromorphone was permitted if needed. Patients were excluded if they had moderate or severe hepatic impairment (defined as aspartate aminotransferase [AST], alanine aminotransferase [ALT], or gamma-glutamyl transpeptidase values that exceeded the normal value by more than fivefold, or serum albumin <20 g/L), or had been administered a drug with a known impact on serum creatinine elimination ≤14 days before study registration. All study participants provided written informed consent before study start.

### Study design

This was an open-label, prospective, parallel-comparison, interventional study conducted at one hospital to determine the effect of renal impairment on the steady-state pharmacokinetics of intravenous hydromorphone in Japanese patients receiving treatment for cancer pain. CLcr values were obtained by urine analysis and used for assignment to one of four groups: CLcr ≥90 mL/min (normal), 60–<90 mL/min (mild impairment), 30–<60 mL/min (moderate impairment), or <30 mL/min (severe impairment). The groups were defined according to guidelines issued by the Food and Drug Administration's Center for Drug Evaluation and Research^22^ and the European Medicines Agency.^23^

Blood and urine were collected to assess various pharmacokinetic parameters. Blood was collected (6 mL/collection) on days one and two (24 hours after day one). Day one samples were used to determine the steady-state concentration (Css) and to calculate pharmacokinetic parameters; day two samples were collected as backup samples. If a rescue dose of hydromorphone was administered before a scheduled blood withdrawal, blood was collected at least three hours after the dose was given. Patients underwent a 24-hour urine collection starting from day one. Urine was collected at the patient's bedside and remained at room temperature throughout the 24-hour collection period. The concentrations of hydromorphone and H3G in plasma and urine were measured using liquid chromatography with tandem mass spectrometry conducted by LSI Medience Corporation (Tokyo, Japan). Uremic toxins (indoxyl sulfate, phenyl sulfate, and p-cresol sulfate) were also measured in the plasma.

The concomitant use of drugs with a known effect on serum creatinine elimination, opioids other than hydromorphone, oral hydromorphone, or nalmefene hydrochloride hydrate, was prohibited during the study. The rescue dosing of hydromorphone was not limited during the study.

This study was conducted in accordance with the Declaration of Helsinki and the Japanese Clinical Trial Act. The study protocol was approved by the Clinical Trial Review Board at Shimane University Hospital (Clinical trial registration: Japan Registry for Clinical trials [jRCT 1061190042]).

### Outcomes

The primary outcome was hydromorphone systemic clearance at steady state (CLss). Secondary pharmacokinetic outcomes included plasma concentrations of hydromorphone and H3G, urinary excretion rate (Fe) and renal clearance (CLrenal) of hydromorphone and H3G, and the ratio of plasma concentrations of H3G to hydromorphone. The relationship between the plasma concentration of each uremic toxin and CLss was an exploratory pharmacokinetic outcome. The safety outcome was adverse events (AEs). AEs were evaluated during the 24-hour study period in patients who were on a continuous dose of hydromorphone. AEs were defined as any unfavorable or unintended sign, symptom, or disease during the 24-hour study period, including worsening of a concomitant disease, regardless of whether or not the AE was related to hydromorphone administration. Symptoms or diseases that continued from the observational period were not considered AEs. AEs were recorded and coded using the Medical Dictionary for Regulatory Activities, version 23.0.

### Statistical methods

The sample size of 48 patients (12 per group) was determined by considering feasibility of patient enrollment. The minimum number of patients (per group) considered necessary to perform statistical analyses was eight.

The measured day one values for Css of hydromorphone and H3G were used for evaluation and pharmacokinetic parameter calculation. Noncompartmental analyses were used to calculate pharmacokinetic parameters for individual patients. Geometric means and standard deviations (SDs) using point estimates, and the 90% confidence interval using the interval estimate, were calculated for the total population and each group. No statistical hypothesis test was performed between groups. The relationships between CLcr and pharmacokinetic parameters were plotted and Pearson's correlation coefficient was calculated. Values obtained from the normal renal function group were used as the standard for comparisons between groups. Because of the small number of patients in the group with normal renal function, additional analysis was performed using the combined data of patients in the normal and mild group as reference.

Logarithmic transformation and back transformation were used to calculate the ratio of each group with renal impairment (mild, moderate, or severe) to the normal group for each pharmacokinetic parameter. Regarding rescue dosing and the Fe calculations, if the ratio of rescue dose to one-day dose during a 24-hour urine collection was >20%, the patient was excluded from the Fe calculation. Statistical analyses were performed using SAS version 9.4 (SAS Institute, Inc., Cary, NC).

## Results

### Patients

Patients were registered between April 2020 and May 2021. Thirty-two patients were enrolled (renal impairment: normal, *n* = 3; mild, *n* = 10; moderate, *n* = 15; and severe, *n* = 4), completed the study, and were included in the statistical analyses. The normal and severe groups did not reach the set minimum of eight patients for statistical analyses, owing to concerns related to the spread of COVID-19. The study was terminated after at least three patients were recruited into each group, which was considered the minimum to make evaluation possible. The study could not be extended further.

Baseline characteristics are shown in Table 1. The mean age was younger in the normal versus other groups. The fixed dose of hydromorphone ranged from 0.5 to 7.4 mg/day; mean daily dose decreased with decreasing renal function. No patient had their hydromorphone dose reduced or increased because of pain control during the study period. Seven patients had a >20% rescue dose to one-day dose ratio during the 24-hour urine collection period and were excluded from the Fe analysis.

**Table 1.** Patient Characteristics and Treatment

	Whole population (*n* = 32)	CLcr ≥90 (*n* = 3)	CLcr 60–<90 (*n* = 10)	CLcr 30–<60 (*n* = 15)	CLcr <30 (*n* = 4)
Sex, *n* (%)					
Male	15 (46.9)	1 (33.3)	7 (70.0)	5 (33.3)	2 (50.0)
Female	17 (53.1)	2 (66.7)	3 (30.0)	10 (66.7)	2 (50.0)
Age (years)					
Mean ± SD	72.2 ± 12.7	55.0 ± 14.0	73.3 ± 11.5	73.8 ± 12.0	76.5 ± 11.3
Min, max	45, 102	45, 71	53, 93	55, 102	61, 88
Weight (kg)					
Mean ± SD	47.27 ± 11.43	51.70 ± 17.45	46.13 ± 9.31	44.94 ± 11.44	55.53 ± 11.56
Min, max	25.7, 70.9	36.8, 70.9	36.0, 61.9	25.7, 66.0	42.8, 66.1
Body mass index (kg/m^2^)					
Mean ± SD	19.02 ± 4.09	19.83 ± 5.20	18.13 ± 3.08	18.54 ± 4.25	22.40 ± 4.74
Min, max	12.1, 28.6	14.7, 25.1	15.3, 25.6	12.1, 28.6	17.7, 27.1
<25, *n* (%)	27 (84.4)	2 (66.7)	9 (90.0)	14 (93.3)	2 (50.0)
Primary disease, *n* (%)					
Esophageal	3 (9.4)	0	2 (20.0)	1 (6.7)	0
Stomach	5 (15.6)	1 (33.3)	0	3 (20.0)	1 (25.0)
Lung	5 (15.6)	1 (33.3)	1 (10.0)	3 (20.0)	0
Pancreatic	4 (12.5)	0	0	3 (20.0)	1 (25.0)
Prostate	2 (6.3)	0	1 (10.0)	0	1 (25.0)
Head and neck	4 (12.5)	0	4 (40.0)	0	0
Kidney	3 (9.4)	0	1 (10.0)	2 (13.3)	0
Cervix	2 (6.3)	0	0	1 (6.7)	1 (25.0)
Other	7 (21.9)	1 (33.3)^a^	2 (20.0)^b^	4 (26.7)^c^	0
Creatinine clearance (mL/min)					
Mean ± SD	55.298 ± 24.530	104.110 ± 9.189	72.792 ± 8.688	42.775 ± 7.167	21.918 ± 4.798
Min, max	17.15, 112.64	94.38, 112.64	60.89, 87.56	32.28, 59.01	17.15, 26.25
Hydromorphone fixed dose (mg/day)					
Mean ± SD	2.77 ± 2.16	4.08 ± 3.15	3.34 ± 1.74	2.54 ± 2.37	1.18 ± 0.24
Min, max	0.5, 7.4	1.2, 7.4	2.0, 7.0	0.5, 7.0	1.0, 1.5

^a^ Colorectal (*n* = 1).

^b^ Hepatocellular (*n* = 1), bladder (*n* = 1).

^c^ Ureteric (*n* = 1), ovarian (*n* = 1), mucinous cystadenocarcinoma of ovary (*n* = 1), vulval (*n* = 1).

SD, standard deviation.

### Pharmacokinetics

**FIG. 1.**
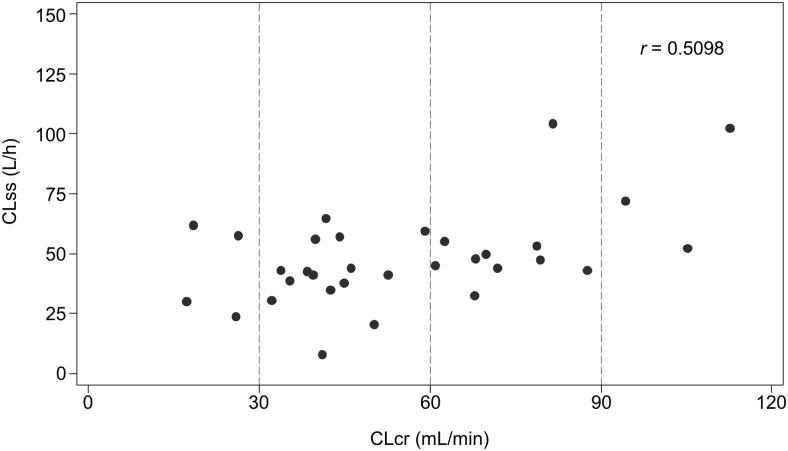
Hydromorphone CLss versus CLcr on day one. CLcr, creatinine clearance; CLss, steady-state clearance; *r*, Pearson's correlation coefficient.

**FIG. 2.**
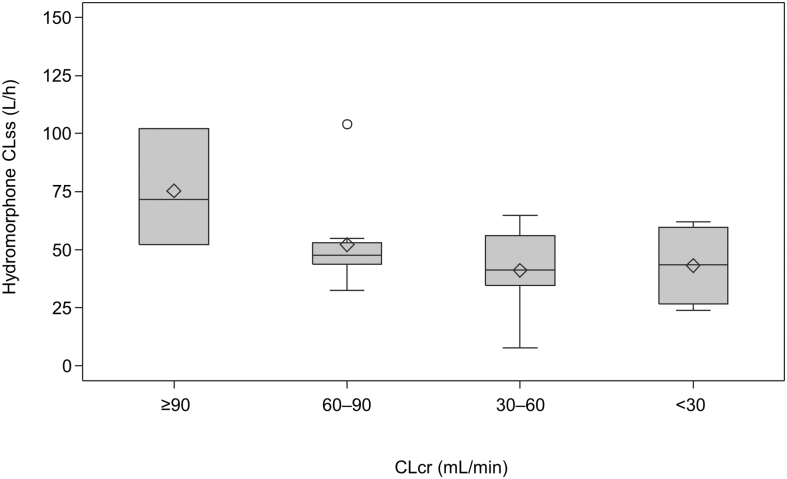
Hydromorphone CLss on day one according to renal function. The horizontal line in the middle of each box indicates median; the ◊ indicates mean (arithmetic mean); the ○ indicates outlier data points located outside the whiskers; and the top and bottom borders of the box indicate upper and lower limits of the interquartile range, respectively. The whiskers above and below the box extend to the data point furthest from the box. CLcr, creatinine clearance; CLss, steady-state clearance.

The scatter plot of the individual CLcr and CLss for hydromorphone revealed that CLss slightly decreased with decreased CLcr until it was <90, after which it remained stable (Pearson's correlation coefficient, *r* = 0.5098; Fig. 1). Box plots of CLss for each group are shown in Figure 2 and hydromorphone and H3G pharmacokinetic parameters are summarized in Table 2. The median CLss values for hydromorphone were reduced in the patients with renal dysfunction versus the normal group. The ratios of the hydromorphone CLss geometric mean values for the mild, moderate, and severe groups to the normal group were 0.69, 0.52, and 0.55, respectively. Results of the additional analysis using normal + mild groups as reference are shown in Supplementary Table S1.

**Table 2.** Pharmacokinetic Parameters

	Whole population (*n* = 32)	CLcr ≥90 (*n* = 3)	CLcr 60–<90 (*n* = 10)	CLcr 30–<60 (*n* = 15)	CLcr <30 (*n* = 4)
Hydromorphone					
CLss (L/h)					
Mean ± SD	48.05 ± 19.69	75.34 ± 25.09	52.16 ± 19.28	41.16 ± 14.88	43.17 ± 19.17
Median (min, max)	44.33 (7.8, 104.2)	71.74 (52.2, 102.0)	47.69 (32.6, 104.2)	41.12 (7.8, 64.7)	43.50 (23.8, 61.9)
Ratio to CLcr ≥90					
Ratio of geometric mean^a^		1 (ref.)	0.69	0.52	0.55
(90% CI)			(0.41, 1.14)	(0.31, 0.84)	(0.30, 1.02)
Css (ng/mL/mg dose)					
Mean ± SD	1.09 ± 0.85	0.60 ± 0.19	0.87 ± 0.22	1.33 ± 1.16	1.14 ± 0.53
Median (min, max)	0.94 (0.4, 5.3)	0.58 (0.4, 0.8)	0.87 (0.4, 1.3)	1.01 (0.6, 5.3)	1.06 (0.7, 1.8)
CLrenal (L/h)					
Mean ± SD	6.40 ± 4.96	17.01 ± 5.66	7.32 ± 4.72	4.74 ± 1.69	2.35 ± 0.94
Median (min, max)	5.26 (1.4, 22.9)	16.60 (11.6, 22.9)	5.66 (3.7, 20.1)	5.00 (1.6, 7.7)	2.34 (1.4, 3.4)
Fe (% of dose)					
Mean ± SD	11.89 ± 4.44	16.20 ± 0.09	13.01 ± 3.78	12.07 ± 3.67	4.99 ± 4.12
Median (min, max)	12.15 (2.4, 20.2)	16.20 (16.1, 16.3)	12.15 (6.7, 16.9)	12.17 (5.9, 20.2)	2.87 (2.4, 9.7)
H3G					
Css (ng/mL/mg dose)					
Mean ± SD	19.21 ± 13.54	6.65 ± 0.78	12.58 ± 3.06	19.53 ± 9.85	43.96 ± 17.13
Median (min, max)	14.95 (5.8, 62.5)	6.83 (5.8, 7.3)	13.07 (7.7, 17.0)	16.31 (8.1, 39.6)	45.97 (21.4, 62.5)
CLrenal (L/h)					
Mean ± SD	2.75 ± 1.47	5.73 ± 0.96	3.61 ± 1.04	2.04 ± 0.40	1.07 ± 0.22
Median (min, max)	2.32 (0.8, 6.8)	5.30 (5.1 6.8)	3.27 (2.3, 5.5)	2.19 (1.4, 2.8)	1.05 (0.8, 1.3)
Fe (% of dose)					
Mean ± SD	55.31 ± 16.58	50.29 ± 6.60	61.37 ± 13.27	49.99 ± 17.53	59.97 ± 25.65
Median (min, max)	54.95 (24.7, 85.6)	50.29 (45.6, 55.0)	65.00 (41.0, 78.0)	50.28 (24.7, 74.0)	60.05 (34.3, 85.6)

^a^ Logarithmic ratio was calculated and converted.

CI, confidence interval; CLrenal, renal clearance; CLss, steady-state clearance; Css, steady-state concentration; Fe, urinary excretion rate; H3G, hydromorphone-3-glucuronide; SD, standard deviation.

**FIG. 3.**
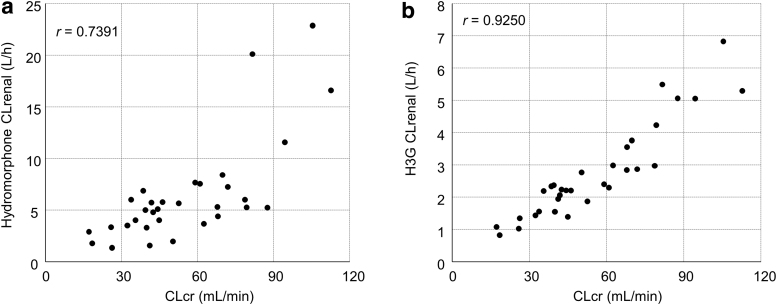
Scatter plots showing the relationship between CLcr and CLrenal on day one for **(a)** hydromorphone and **(b)** H3G. The *r* value indicates the Pearson's correlation coefficient. CLcr, creatinine clearance; CLrenal, renal clearance; H3G, hydromorphone-3-glucuronide.

**FIG. 4.**
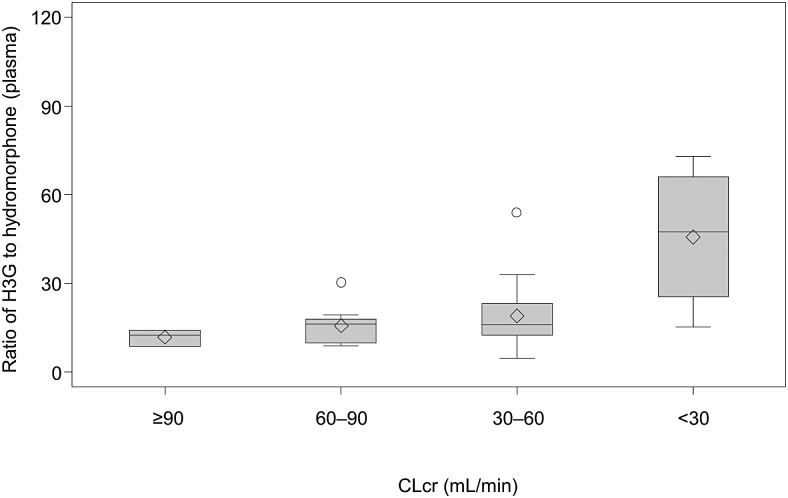
Ratio of H3G to hydromorphone concentration in plasma on day one according to renal function. The horizontal line in the middle of each box indicates median; the ◊ indicates mean (arithmetic mean); the ○ indicates outlier data points located outside the whiskers; and the top and bottom borders of the box indicate upper and lower limits of the interquartile range, respectively. The whiskers above and below the box extend to the data point furthest from the box. CLcr, creatinine clearance; H3G, hydromorphone-3-glucuronide.

The CLrenal correlated with severity of renal impairment for both hydromorphone and H3G (Fig. 3). The mean CLrenal value for hydromorphone decreased with reduced renal function. Similarly, CLrenal for H3G was lower in the mild, moderate, and severe groups versus normal. The ratio of H3G to hydromorphone concentration in the plasma increased with the severity of renal dysfunction, demonstrating that H3G CLrenal decreased with reduced renal function (Fig. 4).

There was no clear correlation between the concentration of any of the uremic toxins and the hydromorphone CLss (Supplementary Table S2**)**.

### Safety

No AE was reported in this study.

## Discussion

This is the first study to assess the effect of renal impairment on the steady-state pharmacokinetics of intravenous hydromorphone in patients with cancer pain. There was a minor difference in the pharmacokinetics of hydromorphone between patients with mild, moderate, or severe renal impairment. The ratio of CLss to the normal renal function group was similar among the mild, moderate, and severe renal dysfunction groups.

In a Phase 2 study of intravenous hydromorphone, which excluded patients with impaired renal function (CTCAE Grade 3 or higher), the mean hydromorphone CLss in Japanese cancer patients during continuous intravenous administration was 51.3 ± 17.4 L/h (mean ± SD, *n* = 28).^24^ Although the CLss for the normal group in our study was higher (75.34 L/h), the mean CLss for normal + mild patients was similar (57.51 ± 22.07 L/h). The Fe up to 48 hours after a single bolus injection was 8.18% ± 2.21% (mean ± SD, *n* = 6) in healthy Japanese participants,^24^ lower than in the normal (16.20%) and normal + mild (13.59%) groups of this study. There was no clear difference in Fe between the normal and normal + mild groups. The normal group included three patients; therefore, hydromorphone pharmacokinetic comparisons between healthy subjects and patients with cancer who have normal kidney function require cautious interpretation.

In our study, hydromorphone CLss decreased 0.52–0.69-fold (ratio calculated using geometric mean values) with renal impairment. In an oral hydromorphone pharmacokinetics study, the ratio of the hydromorphone AUCs for normal renal function and moderate or severe renal impairment was ∼1:2:4, respectively.^18^

There are several differences between our study and previous studies, such as the participants (patients with cancer vs. healthy subjects), administration route (intravenous vs. oral), and continuous administration versus single or multiple administrations; our data provide new insights in that, the urinary excretion rate of hydromorphone was not high and was obtained from patients who actually use hydromorphone for treatment.

Our findings are likely applicable to a range of patients with renal impairment: a clinical pharmacology study found no clear difference in hydromorphone pharmacokinetics between Japanese and Caucasian participants after a single oral administration.^24^ The AUC and maximum concentration ratios (Japanese/Caucasian) for hydromorphone were 1.18 and 1.06, respectively, after 1.3 mg hydromorphone oral administration; those for H3G were 0.99 and 1.17, respectively. Hydromorphone is extensively metabolized by glucuronidation, by variant UGT2B7 hepatic enzymes, although there is no functional (metabolic) difference between alleles.^25^ Therefore, the different *UGT2B7* allele frequencies between Japanese and Caucasians^26^ had little influence on hydromorphone pharmacokinetics.

The urinary excretion rate of hydromorphone was 11.89%, indicating that drug elimination was mainly through drug metabolism. In our study, patients with moderate or worse hepatic impairment were excluded, thus eliminating the influence of impaired hepatic function on drug metabolism. However, two patients with AST levels >60 U/L (one in the mild group and another in the severe group) had H3G concentrations of 25.1 and 21.4 ng/mL, which did not differ substantially from those in other patients, indicating that elevated AST has no notable effect on hydromorphone metabolism. The three patients with liver metastases and one with kidney metastasis were confirmed suitable for inclusion in the analysis based on H3G concentrations, H3G/hydromorphone concentration ratio, and the location of the ratio on a scatter plot. Thus, it appears that hepatic impairment in these three patients had little impact on hydromorphone pharmacokinetics in our study.

Among patients who had a CLcr decrease, the Css increase for H3G was greater than that for hydromorphone. As a result, plasma concentrations of hydromorphone and H3G increased with increased severity of renal impairment. Previous pharmacokinetic studies in healthy Japanese participants found ratios of H3G to hydromorphone concentrations (72 hours post-intravenous administration) ranging from 2.6 to 21.9.^24^ In Babul et al's study, the AUC ratio at steady state after multiple oral administrations was increased to around 100 in one patient with severe renal impairment,^27^ similar to that observed for patients with severe renal impairment in our study.

With normal kidney function, mean urinary excretion rates of hydromorphone and H3G were 16.20% and 50.29%, respectively; kidney function decrease had a greater impact on H3G than hydromorphone. Although the Fe of hydromorphone was decreased in patients with renal impairment versus normal renal function, that of H3G was similar among the groups with renal impairment, which may be explained by hydromorphone metabolism being maintained mostly in patients with renal impairment.

Patients with impaired renal function accumulate uremic toxins,^28^ which inhibit the metabolism of some drugs, including hydromorphone *in vitro*, by inhibiting UGT enzyme activities.^29^ Therefore, uremic toxin accumulation may alter drug metabolism. In our study, no clear correlation was observed between plasma uremic toxin concentration (Supplementary Table S2) and the CLss of hydromorphone (Supplementary Tables S1 and S2). This may have been because the concentration of uremic toxins near the UGT enzymes was not high enough to inhibit hydromorphone metabolism and/or that *in vivo*, uremic toxins do not significantly inhibit UGT enzymes to change the pharmacokinetics of hydromorphone. Further investigation is needed to clarify this correlation.

No AE was reported in our study so we did not obtain any new information regarding hydromorphone safety in patients with renal impairment. H3G is reported to have neuroexcitatory potential, although there are no clinical data available on this effect during long-term hydromorphone therapy.^30^ An *in vivo* study of intracerebroventricular administration of H3G to rats demonstrated a neuroexcitatory effect, which was more potent than that reported for morphine-3-glucuronide, a metabolite of morphine.^31^ However, based on the experimental evidence, hydromorphone appears not to cause AEs when it is used at standard doses in patients with renal failure, and is apparently quite safe in patients who require dialysis.^14^ Nevertheless, such patients should be carefully monitored when receiving intravenous hydromorphone.

### Limitations

This study had several limitations. First, the target enrollment was not achieved in the normal and severe groups. Few patients with normal or severely impaired renal function met the inclusion/exclusion criteria. In addition, owing to the COVID-19 pandemic, the number of hospitalized patients eligible for the study markedly decreased, and while the study period was extended once, it was not extended further because ongoing recruitment was unpredictable and the minimum number of patients for analysis was already registered. Second, our study aimed to assess pharmacokinetics rather than the relationship between pharmacokinetics and efficacy or safety. Finally, further investigation into dose adjustments in patients with impaired kidney function and their effect on hydromorphone pharmacokinetics is needed.

## Conclusions

Systemic clearance of intravenous hydromorphone in steady state decreased 0.52–0.69-fold in cancer patients with renal impairment compared with those with normal renal function. The decreases in CLss were similar between moderate and severe renal impairment. This could be useful for clinicians involved in cancer pain management because it provides information related to the safe administration of intravenous hydromorphone at a dose adapted for patients with renal impairment.

## Supplementary Material

Supplemental data

## Data Availability

The datasets generated during and/or analyzed during this study are available from the corresponding author upon reasonable request. The data are not publicly available because they contain information that could compromise the privacy of study participants.
