# Supplementary material for: Steady-State Pharmacokinetics of Intravenous Hydromorphone in Japanese Patients With Renal Impairment and Cancer Pain
Source: J Palliat Med. 2023 Jun 2;26(6):768–75. doi: 10.1089/jpm.2022.0289 (PMC10278029; doi:10.1089/jpm.2022.0289)
Supplement: Supplementary file 1 [file Supp_TableS1-S2.odt]

# Supplementary Materials

Steady-state Pharmacokinetics of Intravenous Hydromorphone in Japanese Patients with Renal Impairment and Cancer Pain

Toshihiko Nakatani, MD, PhD,1 Kazuhito Shiosakai, MSc,2 Tatsuya Hashimoto, MD, PhD,3 Masao Shionoya, MEng,4 Takaaki Akasaka, PhD,5 Kaoru Toyama, PhD,6 Hitoshi Ishizuka, PhD,6 and Yoji Saito, MD, PhD7

1Department of Palliative Care, Shimane University Faculty of Medicine, Izumo, Japan.

2Data Intelligence Department, 5Primary Medical Science Department, and 6Quantitative Clinical Pharmacology Department, Daiichi Sankyo Co., Ltd., Tokyo, Japan. 

3Department of Anesthesiology, Palliative Care Center, Shimane University Hospital, Izumo, Japan.

4Statistical Analysis Department, Mebix, Inc., Tokyo, Japan. 

7Department of Anesthesiology, Shimane University Faculty of Medicine, Izumo, Japan.

Running title: Hydromorphone PK in renal impairment

Supplementary Table S1. Pharmacokinetic Parameters

|  | Whole population  (N = 32) | CLcr ≥60 (CLcr ≥90 and CLcr 60–<90; Standard) (n = 13) | CLcr 30–<60  (n = 15) | CLcr <30  (n = 4) |
| --- | --- | --- | --- | --- |
| Hydromorphone |  |  |  |  |
| CLss (L/h) |  |  |  |  |
| Mean ± SD | 48.05 ± 19.69 | 57.51 ± 22.07 | 41.16 ± 14.88 | 43.17 ± 19.17 |
| Median (min, max) | 44.33 (7.8, 104.2) | 49.94 (32.6, 104.2) | 41.12 (7.8, 64.7) | 43.50 (23.8, 61.9) |
| Ratio to Standard (CLcr ≥60) |  |  |  |  |
| Ratio of geometric meana |  | 1 (ref.) | 0.69 | 0.73 |
| (90% CI) |  |  | (0.52, 0.91) | (0.42, 1.26) |
| CLrenal (L/h) |  |  |  |  |
| Mean ± SD | 6.40 ± 4.96 | 9.56 ± 6.33 | 4.74 ± 1.69 | 2.35 ± 0.94 |
| Median (min, max) | 5.26 (1.4, 22.9) | 7.25 (3.7, 22.9) | 5.00 (1.6, 7.7) | 2.34 (1.4, 3.4) |
| Fe (% of dose) |  |  |  |  |
| Mean ± SD | 11.89 ± 4.44 | 13.59 ± 3.62 | 12.07 ± 3.67 | 4.99 ± 4.12 |
| Median (min, max) | 12.15 (2.4, 20.2) | 16.13 (6.7, 16.9) | 12.17 (5.9, 20.2) | 2.87 (2.4, 9.7) |
| H3G |  |  |  |  |
| Css (ng/mL/mg dose) |  |  |  |  |
| Mean ± SD | 19.21 ± 13.54 | 11.21 ± 3.73 | 19.53 ± 9.85 | 43.96 ± 17.13 |
| Median (min, max) | 14.95 (5.8, 62.5) | 12.20 (5.8, 17.0) | 16.31 (8.1, 39.6) | 45.97 (21.4, 62.5) |
| CLrenal (L/h) |  |  |  |  |
| Mean ± SD | 2.75 ± 1.47 | 4.10 ± 1.35 | 2.04 ± 0.40 | 1.07 ± 0.22 |
| Median (min, max) | 2.32 (0.8, 6.8) | 3.76 (2.3, 6.8) | 2.19 (1.4, 2.8) | 1.05 (0.8, 1.3) |
| Fe (% of dose) |  |  |  |  |
| Mean ± SD | 55.31 ± 16.58 | 59.35 ± 12.86 | 49.99 ± 17.53 | 59.97 ± 25.65 |
| Median (min, max) | 54.95 (24.7, 85.6) | 54.95 (41.0, 78.0) | 50.28 (24.7, 74.0) | 60.05 (34.3, 85.6) |

aLogarithmic ratio was calculated and converted.

CI, confidence interval; CLcr, creatine clearance; CLrenal, renal clearance; CLss, steady-state clearance; Css, steady-state concentration; Fe, urinary excretion rate; H3G, hydromorphone-3-glucuronide; SD, standard deviation.

Supplementary Table S2. Uremic Toxins

|  | Whole population | CLcr ≥90 | CLcr 60–<90 | CLcr 30–<60 | CLcr <30 |
| --- | --- | --- | --- | --- | --- |
| Indoxyl sulfate (μg/mL) |  |  |  |  |  |
| n | 29 | 3 | 9 | 14 | 3 |
| Mean ± SD | 1.05 ± 1.19 | 0.65 ± 0.42 | 0.76 ± 0.31 | 0.95 ± 0.38 | 2.86 ± 3.55 |
| Median (min, max) | 0.86 (0.2, 6.9) | 0.87 (0.2, 0.9) | 0.66 (0.4, 1.3) | 0.90 (0.3, 1.6) | 1.25 (0.4, 6.9) |
| Phenyl sulfate (μg/mL) |  |  |  |  |  |
| N | 25 | 3 | 4 | 14 | 4 |
| Mean ± SD | 0.88 ± 0.98 | 0.38 ± 0.32 | 0.28 ± 0.23 | 0.92 ± 0.87 | 1.72 ± 1.62 |
| Median (min, max) | 0.44 (0.1, 3.2) | 0.22 (0.2, 0.8) | 0.20 (0.1, 0.6) | 0.60 (0.1, 2.9) | 1.76 (0.1, 3.2) |
| p-cresol sulfate (μg/mL) |  |  |  |  |  |
| N | 27 | 3 | 8 | 13 | 3 |
| Mean ± SD | 5.17 ± 6.57 | 3.67 ± 3.89 | 3.29 ± 2.02 | 3.75 ± 2.44 | 17.88 ± 14.94 |
| Median (min, max) | 2.70 (0.2, 32.0) | 2.61 (0.4, 8.0) | 2.54 (1.7, 7.0) | 3.82 (0.2, 10.0) | 19.40 (2.2, 32.0) |

Concentrations below the lower limit of quantification were treated as missing values.

CLcr, creatine clearance; SD, standard deviation.
